# Expansion and activation of monocytic-myeloid-derived suppressor cell via STAT3/arginase-I signaling in patients with ankylosing spondylitis

**DOI:** 10.1186/s13075-018-1654-4

**Published:** 2018-08-03

**Authors:** Yu-feng Liu, Kun-hai Zhuang, Bin Chen, Pei-wu Li, Xuan Zhou, Hua Jiang, Li-mei Zhong, Feng-bin Liu

**Affiliations:** 1 Guangzhou University of Chinese Medicine, Guangzhou, People’s Republic of China; 2 The First Affiliated Hospital of Guangzhou University of Chinese Medicine, The Lingnan Medicine Research Center, Guangzhou, 510405 People’s Republic of China; 3 Department of Laboratory Medicine, Guangdong Second Provincial General Hospital, Guangzhou, 510317 People’s Republic of China; 4 Department of Hematology Oncology, Guangzhou Medical University, Guangzhou Women and Children’s Medical Center, Guangzhou, 510623 People’s Republic of China

**Keywords:** Ankylosing spondylitis, Myeloid-derived suppressor cells, STAT3/arginase-I signaling, T cell suppression

## Abstract

**Background:**

Ankylosing spondylitis (AS) is a chronic inflammatory rheumatic disease. The dysregulated immune system plays an important role in the pathogenesis of AS. Myeloid-derived suppressor cells (MDSCs) play a key immunoregulatory role in autoimmune arthritis. The aim of this study was to clarify the underlying immunoregulatory mechanism of MDSCs in patients with AS.

**Methods:**

Flow cytometry was used to analyze the phenotype of MDSCs among peripheral blood mononuclear cells (PBMCs) from 46 patients with AS and 46 healthy control subjects. The correlation between MDSC frequency and the disease index of patients with AS was evaluated. A T cell proliferation experiment was used to evaluate the immunosuppressive function of MDSCs.

**Results:**

Polymorphonuclear (PMN) and monocytic (M)-MDSCs were significantly elevated in the PBMCs of patients with AS, when compared with levels in healthy controls. Additionally, M-MDSC levels correlated positively with the clinical index of AS, including the Bath ankylosing spondylitis disease activity index (BASDAI) score, erythrocyte sedimentation rate (ESR) and C-reactive protein (CRP) levels. M-MDSCs derived from patients with AS suppressed T cell responses, and this effect was dependent on the induction of arginase-I. Furthermore, AS-derived M-MDSCs showed high levels of phosphorylated STAT3. Stattic, a STAT3-specific inhibitor, and STAT3-targeted siRNA abrogated the immunosuppressive function of M-MDSCs. Inhibition of STAT3 signaling also resulted in decreased arginase-I activity.

**Conclusions:**

STAT3/arginase-I signaling plays an important role in both the expansion and activation of M-MDSCs in patients with AS. This information may be beneficial in developing novel therapeutic strategies for preventing AS.

**Electronic supplementary material:**

The online version of this article (10.1186/s13075-018-1654-4) contains supplementary material, which is available to authorized users.

## Key messages

- Expansion of M-MDSCs subset in PBMCs derived patients of AS.
- STAT3/arginase-I pathway mediated the expansion of M-MDSC.

## Background

Ankylosing spondylitis (AS) is a chronic inflammatory disease that affects the axial skeleton, causing characteristic inflammatory back pain [1]. The prevalence of different types of spondyloarthritis is 0.5–1.9%, and interaction between a strong genetic component, mainly by specific HLA-B27 subtypes, and bacteria seems to be crucial for the development of the disease [2]. Although there have been significant findings in understanding the pathogenesis of AS, the exact mechanisms have not yet been identified [3, 4]. Clinical therapy and diagnosis are mainly dependent on the radiographic progression of AS [5]. Therefore, understanding the molecular progression of AS would facilitate early diagnosis and treatment during pathogenesis. Immunohistological studies on sacroiliac joint biopsies have shown immune cell infiltrates, including T cells and macrophages, suggesting that both innate and adaptive immune responses could play a role in AS pathogenesis [6]. Further studies determined that gut immunity, T-lymphocyte activation, and peptide processing before HLA class I presentation are involved in the pathogenesis of AS [7]. Studies showing increased frequencies of interleukin (IL)-17-positive CD4^+^ T cells in peripheral blood mononuclear cells (PBMCs) obtained from patients with AS support the fact that T helper (Th)17 cells are involved in the pathogenesis of inflammatory arthritis [8, 9]. Moreover, imbalances in the T lymphocyte subset ratios, Th1/Th2 and Th17/regulatory T (Treg), were demonstrated in patients with AS [10]. These studies collectively indicate that AS progression may be associated with the degree of immune abnormality.

Myeloid-derived suppressor cells (MDSCs) are a heterogeneous population of cells that consists of myeloid progenitor cells and immature myeloid cells (iMCs). In healthy individuals, iMCs generated in the bone marrow quickly differentiate into mature granulocytes, macrophages, or dendritic cells [11]. In pathological conditions, such as in cancer and some autoimmune diseases, a partial block in the differentiation of iMCs into mature myeloid cells results in the expansion of the MDSC population [12]. MDSCs constitute a unique component of the immune system that regulates immune responses in healthy individuals and in the context of various diseases [13, 14]. MDSCs are classified into two major subsets based on their phenotypic and morphological features: polymorphonuclear (PMN)-MDSCs and monocytic (M)-MDSCs [15]. In mice, MDSCs are defined as cells expressing both Gr-1 and CD11b, and are further classified into two subpopulations based on Ly6G and Ly6C: PMN-MDSC (CD11b^+^ Ly6G^+^ Ly6C^lo^) and M-MDSC (CD11b^+^ Ly6G^−^ Ly6C^hi^) [16]. In human PBMCs, the equivalent subsets to PMN-MDSCs and M-MDSCs are defined as HLA-DR^low/−^ CD11b^+^CD33^+^CD14^−^CD15^+^ and HLA-DR^low/−^ CD11b^+^CD33^+^CD14^+^CD15^−^, respectively [17]. MDSCs are characterized by an immunosuppressive phenotype. L-arginine metabolism plays a central role in the immunosuppressive activity of MDSCs. L-arginine can be metabolized by inducible nitric oxide synthase (iNOS or Nos2), generating citrulline and nitric oxide (NO), or can be converted into urea and L-ornithine by arginase [18]. MDSCs expressing arginase-I (ARG1) reduce the availability of L-arginine, which can result in the loss of CD3ζ expression and impaired T cell function [19].

Several recent reports show that MDSCs play crucial roles in the regulation of autoimmune diseases. The MDSC population showed significant expansion in arthritic mice and in patients with rheumatoid arthritis (RA) and produced high levels of inflammatory cytokines [20]. In addition, MDSCs from collagen-induced arthritis (CIA) model mice and patients with RA promoted the polarization of Th17 cells, displaying T cell suppressive ability [21]. Furthermore, the transfer of these CIA mouse-derived MDSCs facilitated disease progression in CIA model mice [22]. These studies collectively show the potential association of MDSCs with autoimmune arthritis disease as well as the therapeutic value of MDSCs. However, the association between MDSCs and AS has not been examined.

In this study, we report that MDSCs showed expansion in patients with AS compared with healthy controls, and the level of M-MDSCs significantly correlated with the AS disease activity index. AS-derived M-MDSCs displayed a T cell suppressive function, which was mediated through the production of arginase-I and activation of STAT3 (signal transducer and activator of transcription 3) signaling. Our study provides novel insights into a valuable role of M-MDSCs in promoting AS pathogenesis and suggests that M-MDSCs represent a potential immune therapeutic target in AS treatment.

## Methods

### Ethics statement

This research was approved by the ethics review board of Guangdong Second Provincial General Hospital. Written, informed consent was provided by each participant and/or their legal guardian.

### Patients

Peripheral blood samples were obtained from 46 AS patients and 46 healthy control subjects. Patients with AS met the modified New York criteria for AS [23]. The Bath Ankylosing Spondylitis Disease Activity Index (BASDAI) score [24] was measured for the majority of patients with AS at the time when the blood samples were obtained. Age, sex, disease duration, erythrocyte sedimentation rate (ESR), and C-reactive protein (CRP) levels were recorded (Table 1).

**Table 1:** Characteristics of the patients with ankylosing spondylitis

Variable	Healthy control	Ankylosing spondylitis
Number of samples (*n*)	46	46
Age (years)	32.3 ± 1.2	23.6 ± 9.8
Gender, male/female (*n*)	22/24	25/21
Duration of disease (years)	–	5.6 ± 3.6
BASDAI score	–	2.76 ± 1.07
ESR mm/hour	9.2 ± 3.5	24.1 ± 12.4
CRP, mg/liter	2.5 ± 1.2	28 ± 17.1
HLA-B27, positive member	–	12

*BASDAI* Bath Ankylosing Spondylitis Disease Activity Index (range 0–10), *CRP* C-reactive protein, *ESR* erythrocyte sedimentation rate

### Reagents and antibodies

RPMI 1640, DMEM, Lipofectamine 2000, FBS, β-ME, penicillin, 5-(and-6)-chloromethyl-2,7-dichlorodihydrofluorescein diacetate, acetyl ester (CM-H2DCFDA), and 5(6)-carboxyfluorescein diacetate succinimidyl ester (CFSE) were obtained from Invitrogen (Grand Island, NY, USA). NW-hydroxy-nor-arginine (NOHA) and L-NG-monomethyl-arginine (L-NMMA) were obtained from Cayman Chemical (Ann Arbor, MI), N-acetylcysteine (NAC) and dimethyl sulfoxide were purchased from Sigma-Aldrich (Merck, Germany). The following anti-human Abs was purchased from Thermo Fisher Scientific (Waltham, MA, USA): CD11b-FITC, CD33-PE, CD14-PE-Cy7, CD15-eFluor450, HLA-DR-PE-Cy5, CD4-PE, CD8-PE-Cy5, CD3-PE-Cy7, and their corresponding isotope controls.

### PBMCs isolation and flow cytometric analysis

PBMC were isolated from whole blood by Ficoll centrifugation and analyzed immediately. The cell phenotype was analyzed by flow cytometer (BD LSR fortessa; BD Biosciences, San Jose, CA, USA), and the data were analyzed with the FlowJo 10.0 software package (TreeStar Inc., Ashland, OR, USA). Data were acquired as the fraction of labeled cells within a live-cell gate set for 50,000 events. A FACS Aria III (BD Biosciences) was used for flow cytometric sorting. The strategy for MDSCs sorting was to gate HLA-DR^-/low^CD11b^+^CD33^int/high^ cells from PBMC. In some experiment, MDSCs were sorted from PBMC; then, the remaining PBMC were used for the T cell proliferation assay.

### T cell proliferation assay

T cell proliferation was evaluated by CFSE dilution. Purified T cells were labeled with CFSE (3 μM; Invitrogen), stimulated with antiCD3/CD28 antibodies (5 μg/ml, Thermo Fisher Scientific), and cultured alone or co-cultured with autologous PMN-MDSCs or M-MDSCs at the indicated ratios. The cells were then stained for surface marker expression with CD4-PE or CD8-PE-Cy5 antibodies, and T cell proliferation was analyzed on a flow cytometer. T cells without stimulation were used as the negative control.

### Arginase enzymatic activity assay

Arginase-I activity was measured in PMN-MDSC lysates, as previously described [13] with slight modifications. Briefly, cells were lysed with 0.1% Triton X-100 for 30 min, followed by the addition of 25 mM Tris-HCl and10 mM MnCl_2_. The enzyme was activated by incubation for 10 min at 56 °C. Arginine hydrolysis was performed by incubating the lysate with 0.5 M l-arginine at 37 °C for 2 h. The urea concentration was measured at 540 nm after the addition of alpha-isonitrosopropiophenone (dissolved in 100% ethanol), followed by heating at 95 °C for 30 min.

### Reactive oxygen species (ROS) production

Cells (5 × 10^5^) were incubated at 37 °C in the presence of 1 μM CMH_2_DCFDA (Thermo Fisher Scientific) for 30 min and were then labeled with fluorescence-conjugated antibodies (Abs) against CD33 and CD11b. The ROS content in PMN-MDSCs was analyzed by flow cytometry.

### Enzyme-linked immunosorbent assay (ELISA)

The production of interferon (IFN)-γ in culture supernatants was determined by ELISA, following the manufacturer’s instructions (R&D Systems, Minneapolis, MN, USA).

### Transwell assays

PMN-MDSCs isolated by flow cytometric sorting were cultured in Transwell inserts (0.4 mm pore size; EMD Millipore, Billerica, MA, USA), and fresh autologous T cells (1 × 10^6^ cells/ml) were cultured in 96-well plates.

### Intracellular staining

Intracellular staining of phosphorylated STAT-3 (pSTAT-3, phospho Tyr705) was performed following the manufacturer’s protocol (Cell Signaling Technology, Beverly, MA, USA). Cells (5 × 10^5^) were fixed with formaldehyde to stabilize the cell membrane, and permeabilized using BD Fix&Perm Solution (BD Biosciences, Franklin Lakes, NJ, USA). After washing, cells were then stained with Alexa Fluor 488-conjugated pSTAT-3 and analyzed by flow cytometry.

### STAT3 inhibition

Stattic, a STAT3-specific small molecule inhibitor (Calbiochem, MilliporeSigma, Burlington, MA, USA) and short interfering (si) RNA targeting *STAT3* (siSTAT3 ID: 116558, Thermo Fisher Scientific) were used to inhibit *STAT3* signaling. Stattic was diluted to 1% in dimethyl sulfoxide (DMSO). PMN-MDSCs were treated with 10 μM Stattic at 37 °C for 24 h. Scrambled and *STAT3*-targeted siRNAs were transduced into PMN-MDSCs cells using lentiviral vectors [25].

### Generation of retrovirus

Two hunderd ninety three T cells were transfected with a mixture of DNA containing 2.5 μg VSVG, 2.5 μg Δ8.2, and scrambled or *STAT3*-targeted siRNA vectors using Lipofectamine 2000 according to the manufacturer’s instructions. Media containing scrambled or *STAT3* siRNA retroviruses were collected 72 h after transfection and filtered through a 0.45 μm pore-size filter.

### Statistics analysis

All data are presented as the mean ± SEM. Clinical and immunological parameters were compared by non-parametric Mann-Whitney *U* tests. For in vitro experiments, statistical analyses were performed using unpaired or paired *t* tests. Correlations between different parameters were analyzed using a Spearman rank test. Statistical tests were performed using GraphPad Prism version 5.0a (GraphPad Software, San Diego, CA, USA) and SPSS Statistics 17.0 (SPSS Inc., Chicago, IL, USA). *P* values of 0.05 or 0.01 were considered significant.

## Results

### Increased frequency of MDSCs in peripheral blood of patients with AS

**Fig. 1:**
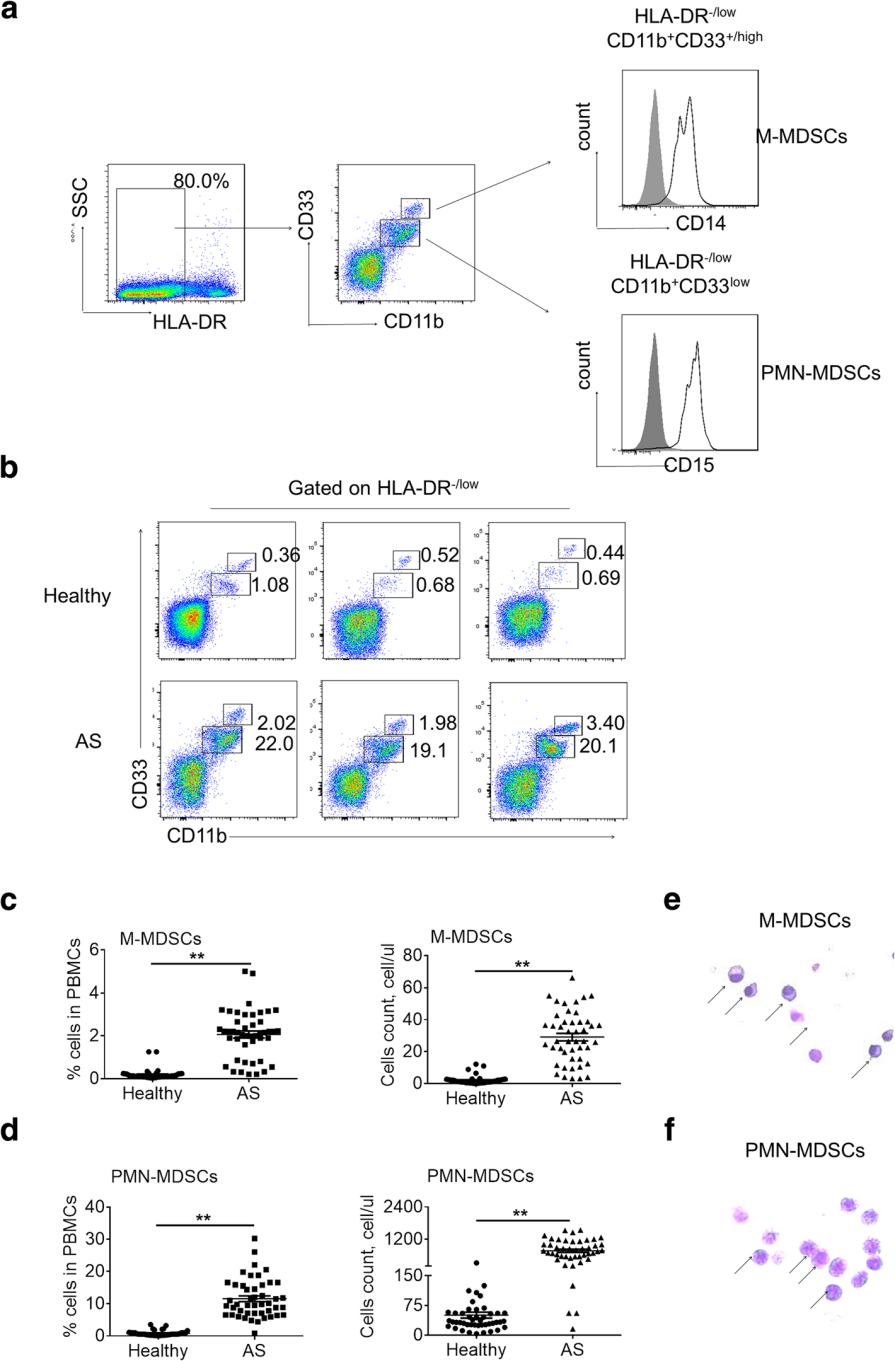
Increased frequency of MDSCs in peripheral blood of patients with AS. **a** Gating strategy of MDSCs by flow cytometry analysis. HLA-DR^−/low^ cells were first selected from live PBMCs, the HLA-DR^–/low^CD11b^int^CD33^int^ and HLA-DR^–/low^CD11b^high^CD33^high^ populations corresponded to the PMN-MDSCs and M-MDSCs subsets, respectively. The expression of cell surface markers CD14^+^ (M-MDSCs) and CD15^+^ (PMN-MDSCs) on this population was subsequently evaluated. *Black*, CD14 or CD15; *gray*, isotype. **b** Representative flow cytometry data for PMN-MDSCs and M-MDSCs from ankylosing spondylitis patients and healthy controls. The *boxed areas* represent the cells percentage in PBMCs, respectively. **c** Statistical analysis of M-MDSCs frequency (*left*) and absolute cell counts (*right*) in the peripheral blood from ankylosing spondylitis patients (*n* = 46) and healthy controls (n = 46). ^**^P<0.01, unpaired *t* test. **d** Statistical analysis of PMN-MDSCs frequency (*left*) and absolute cell counts (*right*) in the peripheral blood from ankylosing spondylitis patients (n = 46) and healthy controls (n = 46). ^**^P<0.01, unpaired *t* test. **e** Wright-Giemsa staining exhibited that M-MDSCs from patients with ankylosing spondylitis showed typical immature cellular morphology. **f** Wright-Giemsa staining exhibited that PMN-MDSCs from patients with ankylosing spondylitis showed typical immature cellular morphology. *AS* ankylosing spondylitis, *M-MDSCs* monocytic myeloid-derived suppressor cells, *PMN-MDSC*polymorphonuclear myeloid-derived suppressor cells

To determine whether MDSCs play a role in patients with AS, using flow cytometry, we first compared the MDSC frequencies and absolute cell counts in the peripheral blood of patients with AS (*n* = 46) with those in age-matched healthy donors (n = 46). The HLA-DR^-/low^CD11b^int^CD33^int^ and HLA-DR^-/low^CD11b^high^CD33^high^ populations corresponded to the PMN-MDSC and M-MDSC subsets, respectively, further based on the expression of CD14 or CD15 (Fig. 1a). By analyzing the MDSC percentage and absolute numbers of PBMCs, we found significant elevations in the frequency (0.1697 ± 0.03879% vs 2.049 ± 0.1810%, *P* < 0.001) and absolute cell counts (1.346 ± 0.1367 vs 28.38 ± 2.516, *P* < 0.001) of M-MDSCs (Fig. 1b–c) and in the frequency (0.8685 ± 0.1229% vs 12.13 ± 0.9299%, *P* < 0.001) and absolute cell counts (52.76 ± 8.316 vs 825.3 ± 57.58, *P* < 0.001) of PMN-MDSCs (Fig. 1b, d) in patients with AS compared with healthy controls. We further assessed the MDSC levels in 18 AS patients receive treatment [anti-tumor necrosis factor (TNF), nonsteroidal anti-inflammatory drugs (NSAIDs) and steroid drugs]. Compared with treatment-naive AS patients, patients who received treatment exhibited lower disease activity (BASDAI, treatment-naive vs treatment: 2.76 ± 1.07 vs 1.091 ± 0.1668) (Additional file 1: Table S1), but still had higher percentages of MDSCs than healthy controls (Additional file 1: Figure S1). Furthermore, we found no significant difference in MDSC levels among the different stages of diseases, including axial disease only and axial plus peripheral disease (Additional file 1: Figure S2). Therefore, we believe that MDSCs may play an important predictive role in early diagnosis of disease. Furthermore, Wright-Giemsa staining showed that the M-MDSCs (Fig. 1e) and PMN-MDSCs (Fig. 1f) from patients with AS exhibited typical immature cellular morphology. These observations collectively demonstrated that the MDSC subset is elevated significantly in the peripheral blood of patients with AS.

### Elevated M-MDSCs correlate with disease index in patients with AS

**Fig. 2:**
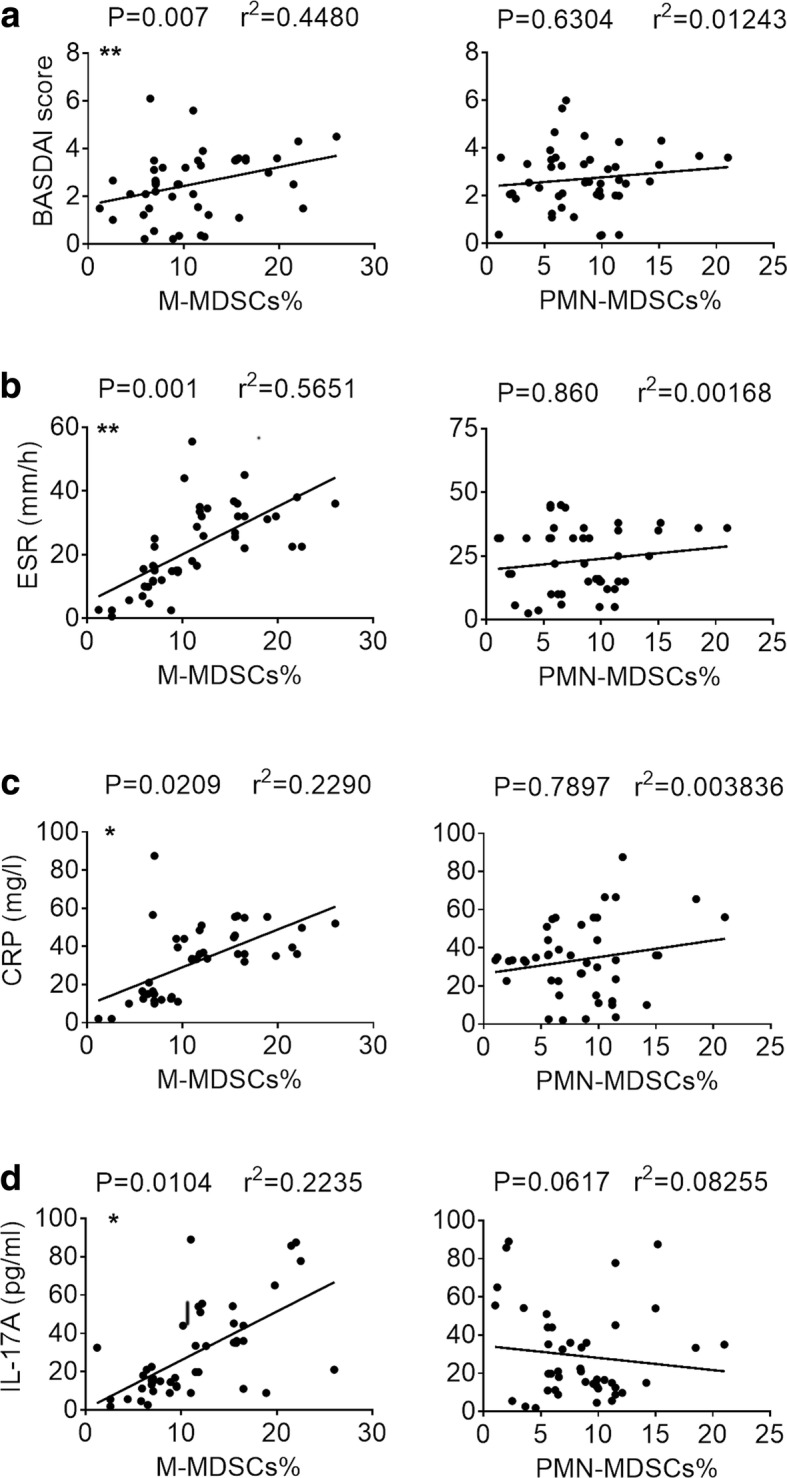
Elevated M-MDSCs correlate with disease index in patients with AS. Correlation between M-MDSCs (*left*) or PMN-MDSCs (*right*) and disease markers in ankylosing spondylitis patients including BASDAI score (**a**), ESR (**b**), CRP (**c**), and concentration of IL-17 (**d**). *CRP* C-reactive protein, *ESR* erythrocyte sedimentation rate, *IL* interleukin, *M-MDSCs* monocytic myeloid-derived suppressor cells, *PMN-MDSC* polymorphonuclear myeloid-derived suppressor cells

To further investigate the clinical significance of increased MDSCs in patients with AS, we evaluated the correlation between MDSC numbers and AS disease index. The BASDAI score is one of a group of classification criteria for spondyloarthropathies and an important predictor of treatment in patients with AS [24]. We found statistically positive correlations between the frequencies of M-MDSCs and the BASDAI scores (*P* = 0.007). In contrast, there was no correlation between the frequencies of PMN-MDSCs and the BASDAI scores (*P* = 0.6304) (Fig. 2a). In addition to BASDAI, further analysis revealed that the frequency of M-MDSCs, but not PMN-MDSCs, correlated positively with the ESR (Fig. 2b) and CRP levels (Fig. 2c) in patients with AS (*P* = 0.001 and *P* = 0.0209, respectively). A previous study reported that PBMCs from patients with AS showed increased numbers of IL-17-positive CD4^+^ T cells compared with control subjects, and the numbers were positively correlated with index of disease activity [9]. Interestingly, we also determined that the levels of M-MDSCs in our study were positively correlated with the concentration of IL-17 (*P* = 0.0104) (Fig. 2d). These results suggested that M-MDSC levels were intimately correlated with disease activity index in patients with AS and indicated that M-MDSCs may represent a novel immunological marker of disease progression in AS.

### M-MDSCs derived from patients with AS suppress T cell responses

**Fig. 3:**
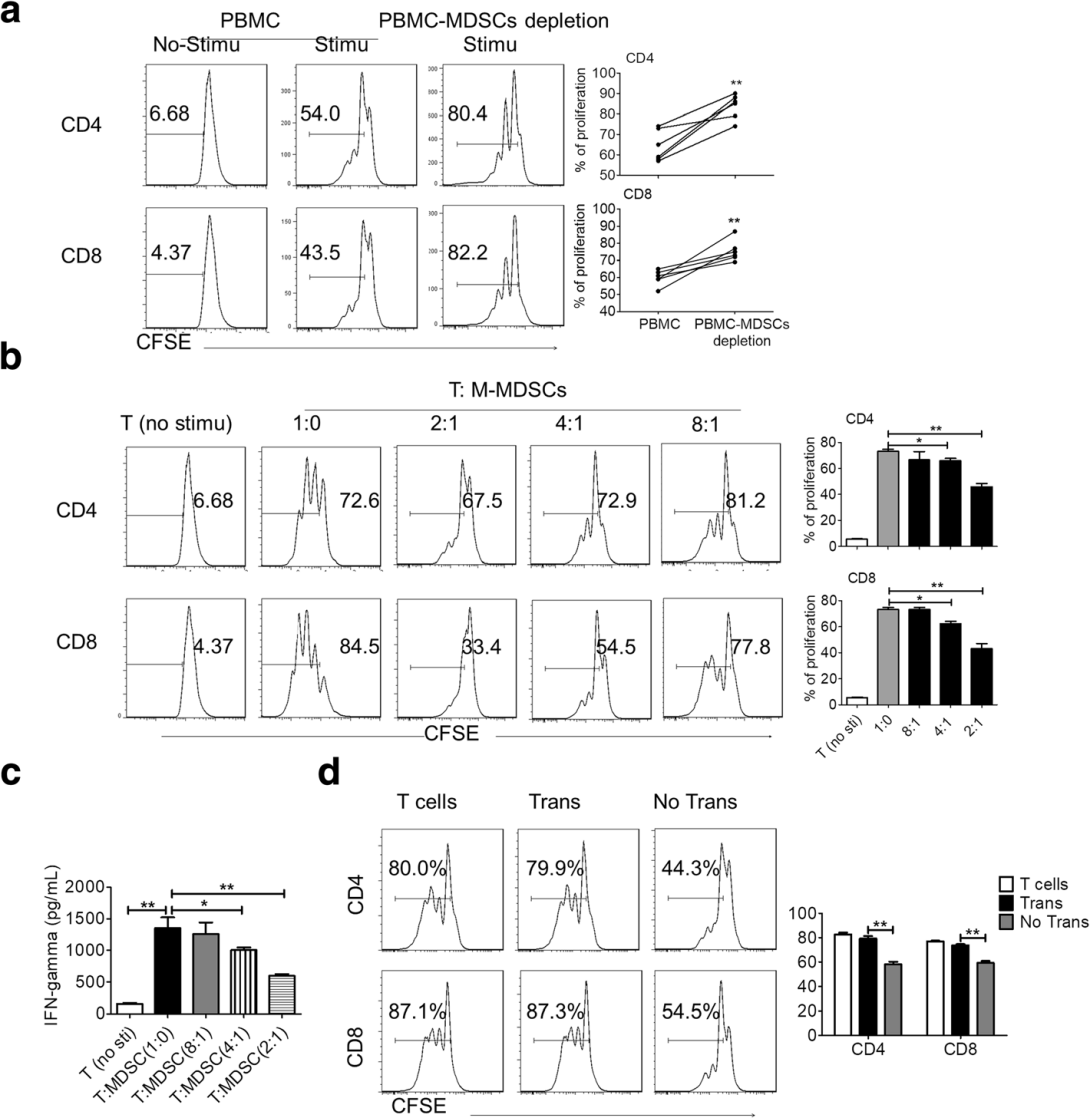
M-MDSCs derived from patients with AS suppress T cell responses. **a** Depletion of MDSCs enhanced T cell function. PBMCs or PBMCs with MDSC depletion (PBMC-MDSC depletion) from fresh peripheral blood of AS patients were stimulated with CD3/CD28, and proliferation of T cells was examined by CFSE dilution. *Left panels*: representative flow cytometry data from one individual; *right* panel: results of stimulated samples from six individuals. **b** CD3^+^ T cell from patients at ankylosing spondylitis patients were stimulated with anti-CD3/CD28, co-cultured with M-MDSCs from the same donors at different ratios for 3d, and T cell proliferation was evaluated by CFSE labeling; unstimulated T cells were used as a negative control. *Left panels*: representative flow cytometry data from one individual; *right panel*: results from five individuals. **c** Production of IFN-γ by T cells in supernatants from panel (**b**) was measured by ELISA. Means and SD are shown; *n* = 6. **d** Co-culture of M-MDSCs-T cell (1:2) experiments as in panel (**b**) were performed, with or without Transwells. ^*^P < 0.05; ^**^P < 0.01, compared with controls by unpaired *t* test. *AS* ankylosing spondylitis, *CFSE*carboxyfluorescein succinimidyl ester, *IFN* interferon, *M-MDSCs* monocytic myeloid-derived suppressor cells, *PMN-MDSC* polymorphonuclear myeloid-derived suppressor cells

MDSCs are known to suppress T cell immune responses under some pathological conditions [26]. Therefore, we evaluated the effect of AS-derived MDSCs on T cell responses. First, MDSCs were depleted from PBMCs-MDSCs by flow cytometric sorting, after which the PBMCs were stimulated with anti-CD3/CD28 antibodies. The results showed that the proliferation of both CD4 and CD8 T cells was enhanced significantly upon MDSC depletion (Fig. 3a). This suggested that the presence of MDSCs in patients with AS suppressed T cell responses. The suppressive activity of MDSCs was further confirmed by co-culture of M-MDSCs or PMN-MDSCs with T cells. M-MDSCs actively suppressed the autologous T cell responses, including cell proliferation (Fig. 3b) and IFN-γ production (Fig. 3c), in a dose-dependent manner. However, PMN-MDSCs from the PBMCs of patients with AS did not suppress T cell responses (Additional file 1: Figure S3A–B). Secondly, to determine whether M-MDSCs function through direct contact with T cells, M-MDSC/T cell co-culture experiments were performed using Transwells. The separation of M-MDSCs from T cells eliminated their suppressive activity (Fig. 3d), demonstrating that the function of M-MDSCs is cell contact-dependent. Our observations from this series of experiments demonstrated that M-MDSCs present in patients with AS actively suppressed T cells in a cell contact-dependent manner.

### AS-derived M-MDSCs suppress T cell responses in an arginase-I-dependent manner

**Fig. 4:**
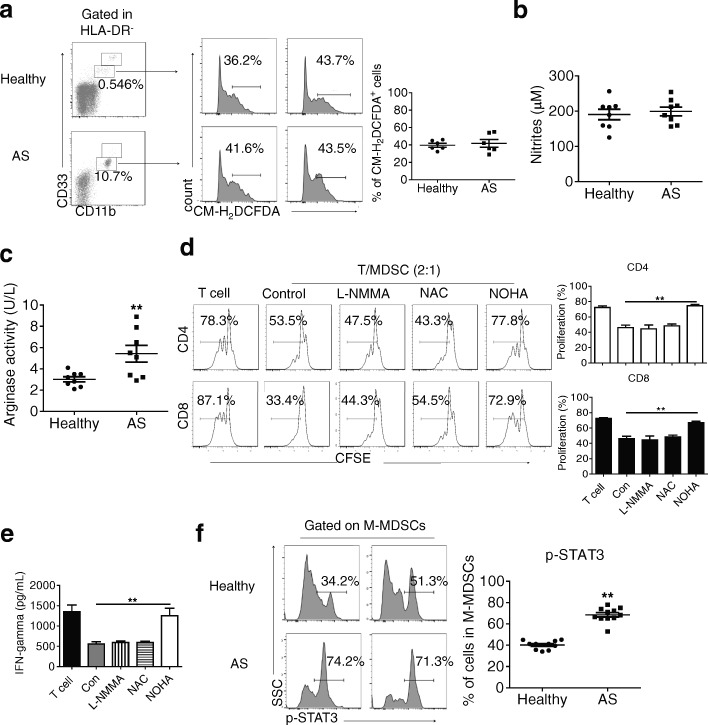
AS-derived M-MDSCs suppress T cell responses in an arginase-I dependent manner. **a** ROS levels in M-MDSCs from patients with ankylosing spondylitis or healthy controls (n = 6) were measured by flow cytometric analysis (*left*). HLA-DR^–/low^CD11b^int^CD33^int^ cells were first gated, and the percentage of CM-H_2_DCFDA^+^ cells is shown. Both representative results (*left*) and means ± SEM from three independent experiments (*right*) are included. **b** NO content in plasma in sorted M-MDSCs from ankylosing spondylitis patients and healthy controls. **c** Arginase activity in M-MDSCs from healthy controls (n = 6) and from patients at ankylosing spondylitis. **d** Effects of different inhibitors on the suppressive function of M-MDSCs from ankylosing spondylitis patients were evaluated by allogeneic mixed lymphocytes reaction. T cells were labeled with carboxyfluorescein succinimidyl ester (CFSE) and stimulated with anti-CD3/anti-CD28 (5 ng/ml). These cells were then co-cultured with M-MDSCs from the same donor at a 2:1 ratio with treatments as indicated for 3 days, and T cell proliferation was evaluated by a flow cytometry. N-hydroxy-L-arginine (NOHA) (an arginase inhibitor, 100 mM); L-NG-monomethyl-L-arginine [L-NMMA, an inducible nitric oxide synthase (iNOS) inhibitor, 100 mM]; N-acetylcysteine (NAC, a ROS inhibitor, 1 mM). **e** Production of interferon (IFN)-γ by T cell supernatants from (**d**) was measured by enzyme-linked immunosorbent assay (ELISA). **f** p-STAT3 levels in M-MDSCs from ankylosing spondylitis patients (*n* = 11) and the control population from healthy donors (*n* = 11) were measured by flow cytometric analysis. *AS* ankylosing spondylitis, *CFSE*carboxyfluorescein succinimidyl ester, *IFN* interferon, *L-NMMA* L-NG-monomethyl-L-arginine, *M-MDSCs* monocytic myeloid-derived suppressor cells, *NAC* N-acetylcysteine, *NOHA* N-hydroxy-nor-L-arginine

Based on the observation that M-MDSCs derived from patients with AS could suppress T cell responses, we further explored the underlying mechanisms controlling M-MDSC-mediated T cell suppression. Inhibition of l-arginine is believed to mediate the immunosuppressive effect of MDSCs under certain pathological conditions [11]. Therefore, we measured the arginase activity, NO content, and ROS levels in M-MDSCs derived from patients with AS and healthy controls. No significant changes were observed in ROS levels (Fig. 4a) or NO content (Fig. 4b). A significant increase in arginase activity was observed in AS-derived M-MDSCs compared with healthy controls (Fig. 4c). To further test this possibility, M-MDSC/T cell co-culture experiments were performed in the presence of different inhibitors, including L-arginine-metabolizing enzymes (*N*-hydroxy-nor-l-arginine, NOHA). The results showed that the suppression of T cell proliferation (Fig. 4d) and IFN-γ production (Fig. 4e) in the presence of M-MDSCs were almost completely recovered after the administration of the arginase inhibitor NOHA, while no similar effects were observed for the NOS inhibitor L-NMMA or the ROS inhibitor *N*-acetylcysteine (NAC) (Fig. 4d–e). Given the functional significance of STAT3 signaling in myeloid cell [27], we analyzed the intracellular phosphorylated STAT3 (pSTAT3) expression level in AS-derived M-MDSCs. AS-derived M-MDSCs had significantly higher expression of pSTAT3 in comparison with healthy control (Fig. 4f). These results demonstrated that AS-derived M-MDSCs suppress T cells in an arginase-dependent manner.

### Inhibition of pSTAT3/arginase-I signaling abrogates the suppressive activity of AS-derived M-MDSCs

**Fig. 5:**
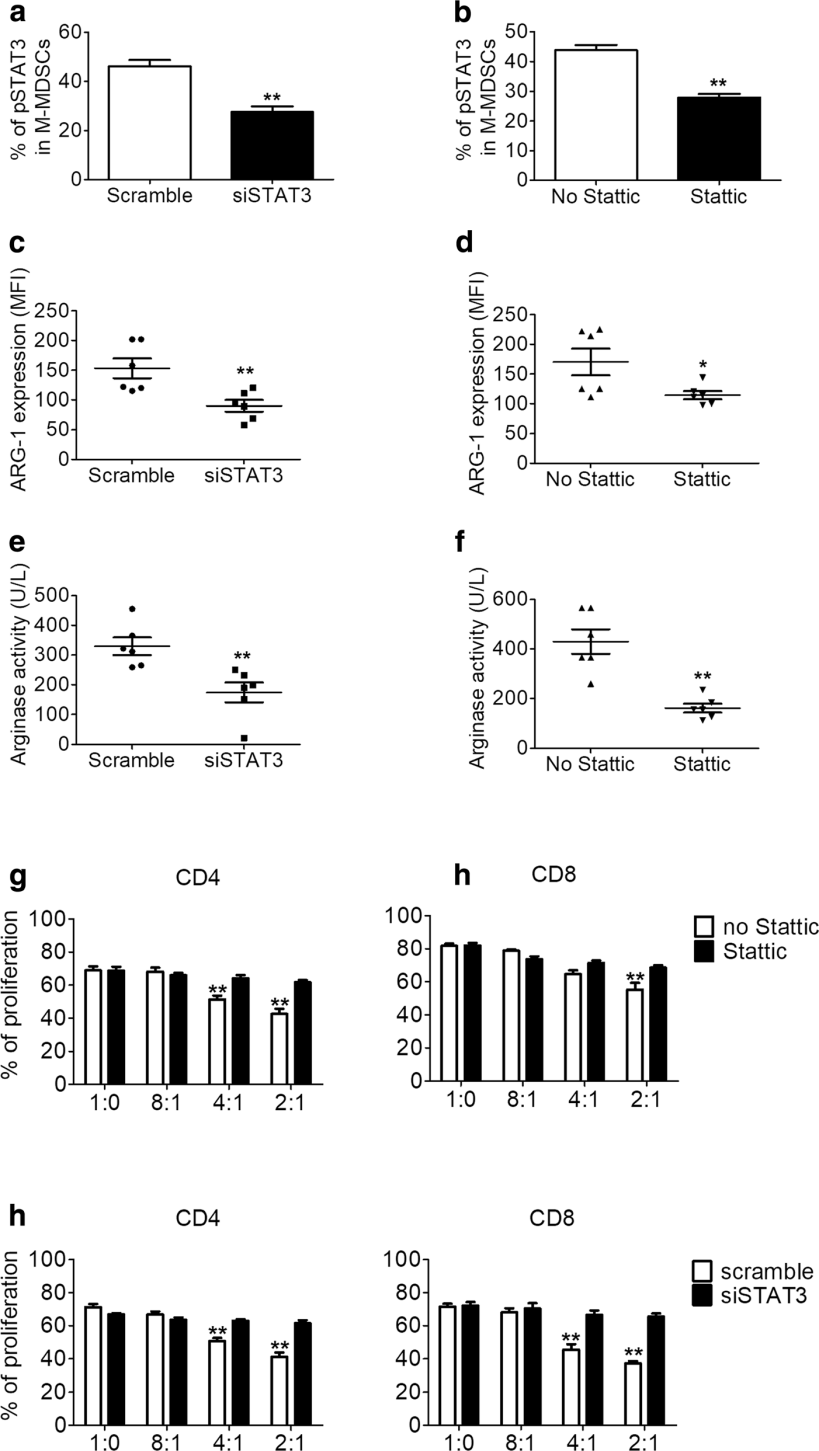
Inhibition of pSTAT3- arginase-I signaling abrogates the suppressive activity of AS-derived M-MDSCs. **a** Inhibition of siSTAT3 signaling on M-MDSCs by scramble or siSTAT3 appropriately decreased the level of pSTAT3 (^*^P < 0.05). **b** Inhibition of STAT3 signaling on M-MDSCs by Stattic (10 μM) appropriately decreased the level of pSTAT3. Intracellular level of ARG1 is decreased with two independent methods of STAT3 signaling inhibition, including siSTAT3 (**c**) and Stattic (**d**). *y* axis shows MFI (^*^P < 0.05). ARG1 activity of ankylosing spondylitis patients derived M-MDSCs after pSTAT3 inhibition with siSTAT3 (**e**) and Stattic (**f**). Inhibition of STAT3 signaling ablates the suppressive activity of M-MDSCs from ankylosing spondylitis patients. Both pSTAT3 small molecule inhibitor (Stattic) (**g**), and STAT3 siRNA (**h**) were able to block the functional suppressive capability of ankylosing spondylitis patients derived M-MDSCs. ^*^P < 0.05; ^**^P < 0.01, compared with controls by unpaired *t* test *ARG1* arginase-I, *M-MDSCs* monocytic myeloid-derived suppressor cells, *pSTAT* phosphorylated signal transducer and activator of transcription 3

Increased levels of pSTAT3 in tumors have been correlated with the increased suppressive activity of tumor-infiltrating MDSCs in patients with cancer [28]. Therefore, we directly investigated the significance of STAT3 signaling in M-MDSCs in patients with AS by inhibiting STAT3 signaling using two independent methods: we used either Stattic, a specific small molecule inhibitor of pSTAT3, or siRNA suppression via lentiviral vector to inhibit STAT3 signaling. Our results showed that both STAT3 signaling inhibition methods appropriately decreased the level of pSTAT3 in M-MDSCs (Fig. 5a–b). Interestingly, both methods of STAT3 signaling inhibition also affected the expression of ARG1 in M-MDSCs (Fig. 5c–d). After treatment with Stattic or siRNA, we found significant decreases in ARG1 activity in AS-derived M-MDSCs (Fig. 5e–f). These data suggest that STAT3 may regulate ARG1 in AS-derived M-MDSCs. Furthermore, both inhibition methods showed that the inhibition of STAT3 signaling abrogated the T cell suppressive function of M-MDSCs derived from patients with AS (Fig. 5g–h). Collectively, these results determined that STAT3 inhibition decreased the ARG1 level and the suppressive activity in AS-derived M-MDSCs.

## Discussion

Although MDSCs have been intensively investigated in autoimmune arthritis, recent studies have shown an emerging role for MDSCs in the pathogenesis of RA [20]. However, the mechanisms for the aberrant expansion of MDSCs in AS as well as their immunological and clinical significance remain unclear. Delineating these important issues will advance our understanding of the relationship between MDSCs and AS, which will benefit the development of immunotherapies to treat human AS diseases.

Here, we report a significant elevation of both subsets of M-MDSCs and PMN-MDSCs in the peripheral blood of patients with AS. Consistent with the CIA mice model and patients with RA, MDSCs significantly expanded in arthritic mice and patients with RA, suggesting that MDSCs play a key role in autoimmune arthritis. However, the mechanism underlying the MDSC expansion in autoimmune arthritis has not yet been determined. In CIA mice, inflammatory cytokines promote myelopoiesis by stimulating the production of myeloid precursors in the bone marrow. Increased levels of TNF-α and/or granulocyte-macrophage colony-stimulating factor (GM-CSF) may promote MDSC accumulation in the PBMC and spleen. In addition, interleukin 6 (IL-6) and transforming growth factor-beta 1 (TGFβ1) genes most likely cause the expansion of MDSCs in inflammatory or cancerous conditions [29]. Similarly, MDSCs from arthritic mice also express higher levels of inflammatory cytokines (e.g. TNF-α, IL-1β) than those from control mice, supporting an inflammatory activation in these cells. These studies suggest that in inflamed tissues, pro-inflammatory cytokines could promote MDSC aggregation, and in turn, MDSCs can releases more pro-inflammatory factors to aggravate inflammatory responses. Moreover, MDSCs from CIA mice and patients with RA promoted the polarization of Th17 cells in vitro [22]. Interestingly, Th17 cells are also involved in the pathogenesis of AS [9]. In our study, we also found that there is a positive correlation between the percentages of M-MDSCs and IL-17 levels in patients with AS. In addition to IL-17, circulating M-MDSCs are elevated significantly in patients with AS with positive correlations to elevated disease activity index, including BASDAI, ESR, and CRP. These data suggest that MDSCs play crucial roles in the regulation of AS.

MDSC-mediated suppression of T cell responses could be beneficial in pathological conditions characterized by the unopposed activation of the immune system such as autoimmune diseases [11]. However, the therapeutic potential of MDSCs in autoimmune arthritis is contradictory. One study showed that the transfer of MDSCs derived from CIA mice and patients with RA facilitated disease progression [30]. In contrast, another study found that the adoptive transfer of CIA-derived MDSCs could reduce the severity of CIA, and the number of Th17 cells also decreased [31]. These inconsistent outcomes may arise from the heterogeneity of MDSCs, factors within the autoimmune inflammatory environment, and different states of the disease. Further investigations are required to study the therapeutic effect of MDSCs in an AS model through AS-derived-M-MDSC transfer, which will be beneficial in understanding the pathological mechanism of AS and provide key insights for developing MDSC-based therapies to treat AS.

MDSCs are primarily defined by their suppressive function [32], however, the functions of MDSCs in patients with AS remain unclear. The T cell suppressive effect of MDSCs in malignant tumors is due to M-MDSCs rather than PMN-MDSCs [33]. However, PMN-MDSCs have been reported to show suppressive functions in autoimmune disease models [34]. It is possible that the phenotypes of MDSCs responsible for suppressing T cell functions differ between tumors and autoimmune diseases. In our study, we found that the T cell suppressive effect of MDSCs in patients with AS is due to M-MDSCs rather than PMN-MDSCs. We examined regulatory factors in M-MDSCs that could control its suppressive function. A critical pathway in tumors and periphery is mediated by STAT3 signaling in the M-MDSC population [35]. In murine models, pSTAT3 regulates the expansion of MDSCs; however, STAT3 has not been reported to directly regulate the T cell suppressive function of MDSCs [11]. In contrast, the immunosuppressive activity of human MDSCs derived from patients with cancer was found to be STAT3-dependent [36]. In this study, we demonstrated that STAT3 signaling plays a functional role in M-MDSCs derived from patients with AS by mediating their ability to suppress autologous T cell proliferation. STAT3 inhibition decreased the level of arginase-I and the T cell suppressive activity in AS-derived M-MDSCs. We also demonstrated that both siRNA suppression and pSTAT3 inhibition using a STAT3-specific inhibitor could abrogate the T cell suppressive function of AS-derived M-MDSCs as well as decrease the level and activity of arginase-I. These results demonstrate that STAT3 signaling is upstream of the arginase-I activity that mediates the suppression of T cell proliferation in patients with AS.

Several STAT3-dependent genes have been reported to play critical roles in M-MDSC function, indicating that there may be multiple pathways of STAT3-dependent immunosuppression. For instance, STAT3-dependent C/EBPβ transcription factor is critical in regulating immunosuppression [37]. HIF1α, another STAT3-dependent gene, mediates the differentiation into tumor-infiltrating macrophages [38]. Further investigations will be beneficial in testing some of these STAT3-dependent pathways in M-MDSCs.

## Conclusions

We report that M-MDSCs were significantly elevated in patients with AS. M-MDSC numbers positively correlated with the AS disease index. The STAT3/arginase-I signaling pathway drove the expansion of M-MDSCs, and mediated the activation of the T cell suppressive function in AS-derived M-MDSCs. Our results collectively suggest that M-MDSCs play an important immunoregulatory role in patients with AS, and therapeutic approaches directed against M-MDSCs may lead to the alleviation of this disease.

## Additional file

Additional file 1: **Figure S1.** MDSC level in the treatment or treatment-naïve AS patients. **Figure S2.** MDSC level in the different stage of AS patients. **Figure S3.** Suppress function assays of PMN-MDSCs derived from patients with AS. **Table S1.** Clinical treatment characteristics of the AS patients. (PDF 399 kb)

## Abbreviations

ARG1: Arginase-I
AS: Ankylosing spondylitis
BASDAI: Bath Ankylosing Spondylitis Disease Activity Index
CFSE: Carboxyfluorescein succinimidyl ester
CIA: Collagen-induced arthritis
CRP: C-reactive protein
ELISA: Enzyme-linked immunosorbent assay
ESR: Erythrocyte sedimentation rate
IL: Interleukin
iMCs: Immature myeloid cells
INF: Interferon
L-NMMA: L-NG-monomethyl-L-arginine
M-MDSCs: Monocytic myeloid-derived suppressor cells
NAC: N-acetylcysteine
NOHA: N-hydroxy-nor-L-arginine
PBMCs: Peripheral blood mononuclear cells
PMN-MDSC: Polymorphonuclear myeloid-derived suppressor cells
RA: Rheumatoid arthritis
ROS: Reactive oxygen species
STAT3: Signal transducer and activator of transcription 3
Th: T helper
Tregs: Regulatory T cells
