# Supplementary material for: Expansion and activation of monocytic-myeloid-derived suppressor cell via STAT3/arginase-I signaling in patients with ankylosing spondylitis
Source: Arthritis Res Ther. 2018 Aug 3;20:168. doi: 10.1186/s13075-018-1654-4 (PMC6091075; doi:10.1186/s13075-018-1654-4)
Supplement: Supplementary file 1 — Figure S1. MDSC level in the treatment or treatment-naïve AS patients. Figure S2. MDSC level in the different stage of AS patients. Figure S3. Suppress function assays of PMN-MDSCs derived from patients with AS. Table S1. Clinical treatment characteristics of the AS patients. (PDF 399 kb) [file 13075_2018_1654_MOESM1_ESM.pdf]

Additional file

Fig 1 **MDSC level is in the treatment or treatment-naïve AS patients.**

Representative flow cytometry data for PMN-MDSCs and M-MDSCs from ankylosing spondylitis patients (Treatment-naive or Treatment) and healthy controls. The boxed areas represent the cells percentage in PBMCs, respectively (Right). Statistical analysis of M-MDSCs and PMN-MDSCs frequency and absolute cell counts in the peripheral blood from AS patients (Treatment-naive or Treatment) and healthy controls. **,P＜0.01, unpaired t test.

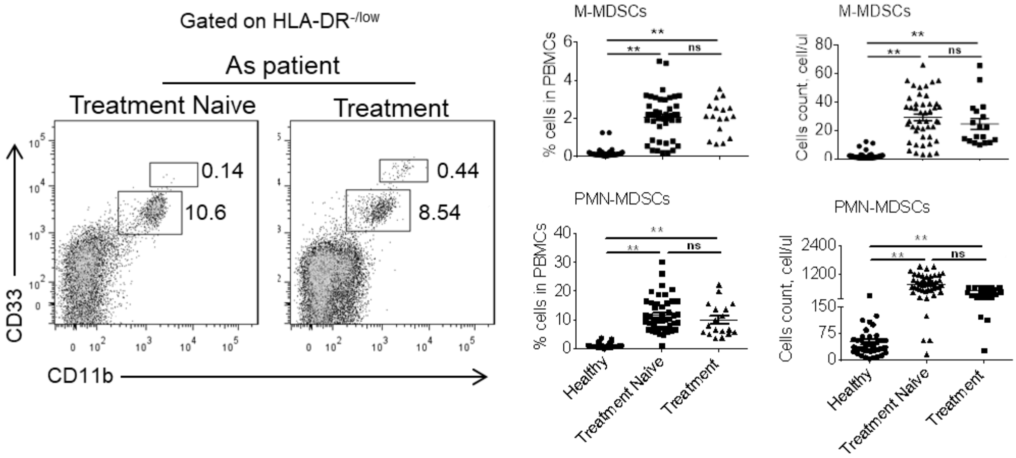

Fig 2 MDSC level is in the different stage of AS patients.

Statistical analysis of M-MDSCs and PMN-MDSCs frequency and absolute cell counts in the peripheral blood from axial only patients (n=34) or axial+PB (n=12) patients.

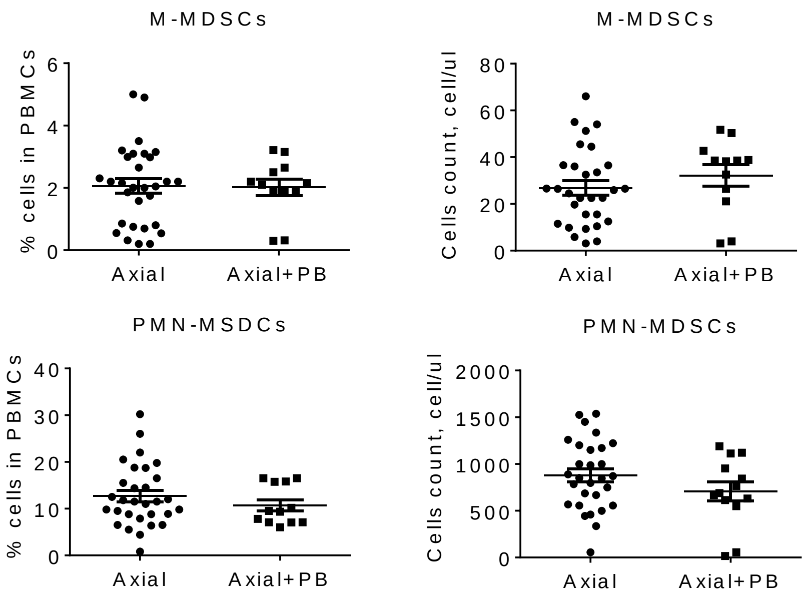

Fig ~~1~~ 3: Suppress function assays of PMN-MDSCs derived from patients with AS: (A) CD3+ T cell from patients at ankylosing spondylitis patients were stimulated with anti-CD3/CD28, co-cultured with M-MDSCs from the same donors at different ratios for 3 d, and T cell proliferation was evaluated by CFSE labeling; unstimulated T cells were used as a negative control. Left panels: representative flow cytometry data from one individual; right panel: results from 5 individuals. (B) Production of IFN-Ɣ by T cells in supernatants from panel B was measured by ELISA. Means and SEM are shown; n=6. *P<0.05; **P<0.01, compared with controls by unpaired *t* test.

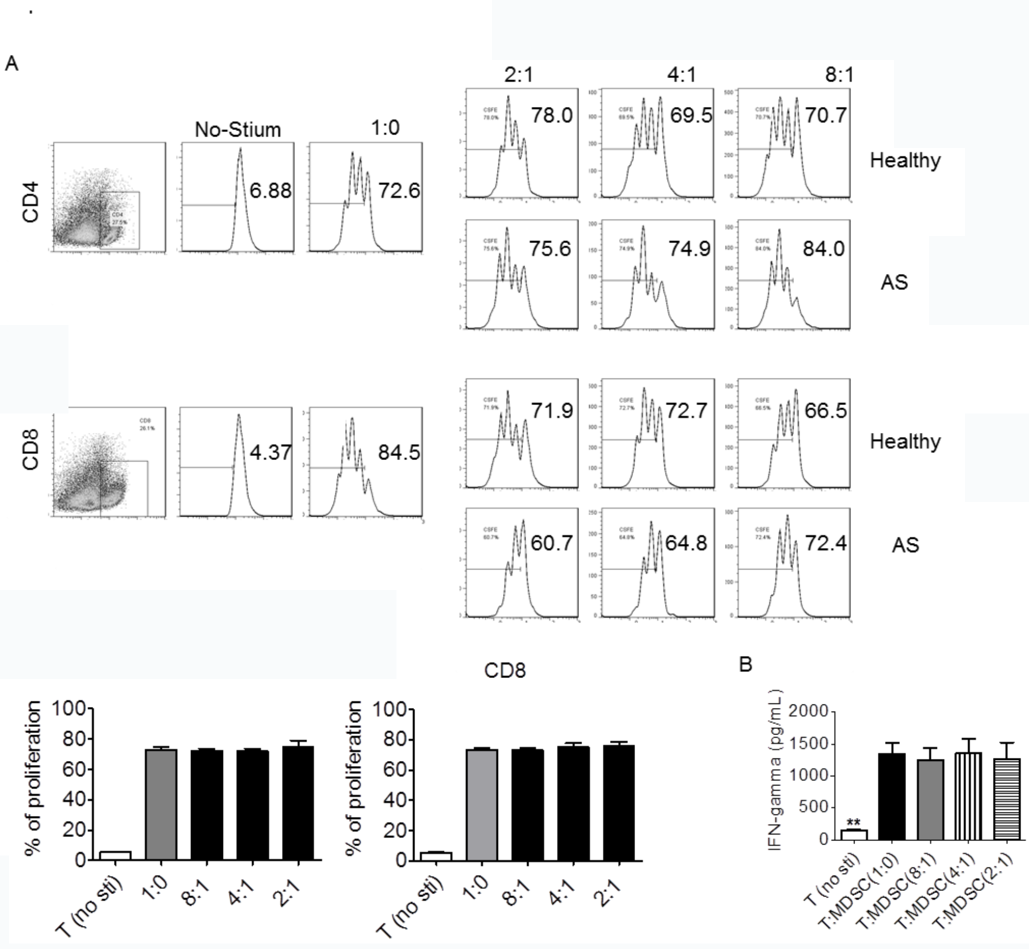

Table 1. Clinical treatment characteristics of the AS patients

Diagnosis, patient	Age/sex	BASDAI	ESR mm/hour	CRP mm/hour	HLA-B27	Treatment
1	43/M	1.1	12	11	-	NSAID,
2	35/M	1.09	9	9	-	Prednisone, SSZ, NSAID
3	44/F	0.2	4	5	+	NSAID, MTX,
4	38/M	0.3	6	6	-	Prednisone, NSAID
5	25/M	0.98	11	12	-	NSAID, MTX
6	39/M	1.2	15	8	+	NSAID, Pred.
7	55/M	2.2	16	6	-	Prednisone, NSAID
8	49/F	1.9	5	7	+	NSAID, SSZ
9	45/M	1.8	11	9	+	MTX, Etan.
10	35/F	2.4	9	5	-	NSAID, Ada., Pred.
11	31/F	2.12	5	6	-	MTX, Etan.
12	33/M	0.56	8	5	-	NSAID, MTX,
13	38/F	0.36	7	3	-	MTX, Etan.
14	41/M	0.55	14	2	+	NSAID, SSZ, Pred.
15	44/F	0.65	10	5	+	NSAID, SSZ
16	49/F	0.45	35	7	-	MTX, Etan.
17	45/M	0.65	26	5	+	NSAID, SSZ
18	55/F	1.12	8	6	-	NSAID, Ada
Average ± SEM	41.33 ± 1.884	1.091 ± 0.1668	11.722±1.836	6.500 ± 0.6009		

BASDAI=Bath Ankylosing Spondylitis Disease Activity Index (range 0-10);
NSAIDs= nonsteroidal anti-inflammatory drugs;
ESR=erythrocyte sedimentation rate; CRP=C-reactive protein;
Anti-TNF treatment: Ada.=adalimumab, Etan.=etanercept;

DMARDs (disease-modifying antirheumatic drugs): MTX=methotrexate, SSZ=sulfasalazine;
Steroid: Pred.=prednisone (10 mg/day);
